# Study of the binding mechanism of aptamer to palytoxin by docking and molecular simulation

**DOI:** 10.1038/s41598-019-52066-z

**Published:** 2019-10-29

**Authors:** Bo Hu, Rong Zhou, Zhengang Li, Shengqun Ouyang, Zhen Li, Wei Hu, Lianghua Wang, Binghua Jiao

**Affiliations:** 1 Department of Biochemistry and Molecular Biology, College of Basic Medical, Second Military Medical University, Shanghai, 200433 China; 2 Marine Biological Institute, College of Marine Military Medicine, Second Military Medical University, Shanghai, 200433 China; 3 Chengdu FenDi Technology Co., Ltd, Chengdu, 610041 China

**Keywords:** Computational models, Computational biophysics

## Abstract

This paper provides a feasible model for molecular structure analysis and interaction mechanism of aptamer and micromolecule. In this study, modeling and dynamic simulation of ssDNA aptamer (P-18S2) and target (Palytoxin, PTX) were performed separately. Then, the complex structure between DNA and PTX was predicted, and docking results showed that PTX could combine steadily at the groove’s top of DNA model by strong hydrogen-bonds and electrostatic interaction. Thus, we truncated and optimized P-18S2 by simulating. At the same time, we also confirmed the reliability of simulation results by experiments. With the experimental and computational results, the study provided a more reasonable interpretation for the high affinity and specific binding of P-18S2 and PTX, which laid the foundation for further optimization and development of aptamers in molecular diagnostics and therapeutic applications.

## Introduction

Palytoxin (PTX) is one of the most powerful natural nonprotein toxin, which is first separated from soft corals in 1971^1^. The LD50 of PTX after intraperitoneal injection is 25 ng/kg and 50 ng/kg in rabbits and mice respectively^2^. Furthermore, PTX can cause human poisonings, such as dizziness, weakness, muscle pain, breathing difficulties, heart failure, and even death, all these poisonings are often associated with ingesting seafood contaminated PTX or direct contact with aerosolized water during dinoflagellate blooms^3–5^. Fortunately, PTX has attracted great attention due to the problem caused by shellfish contamination which harm human health and global shellfish industry development. Many detection methods of PTX have been developed, such as mouse bioassay^6^, liquid chromatography coupled with the fluorometer, ultraviolet-visible spectrophotometer and mass spectrometer^7–9^. However, there are still many challenges, such as ethical issues and expensive instruments. In recent years, we have been working on PTX research and successfully obtained a DNA aptamer named P-18S2 (GGTGGGTCGGACGGGGGTGG), which can bind to PTX with high affinity and specificity, and could serve as a molecular recognition element in diagnosis and biological activity inhibition assays for PTX^10^.

Aptamers are functional single-stranded DNA or RNA oligo nucleotides, which are selected from a random oligonucleotide library through systematic evolution of ligands by the exponential enrichment (SELEX) technique^11^. These aptamers have been isolated and adopted as diagnostic or therapeutic tools, and can bind to various targets with high affinity and specificity by folding into steady and particular three-dimensional structures through intermolecular interactions, such as stacking of aromatic rings, static electricity, van der Waals interaction, hydrogen bond and induced fit mechanism^12,13^. The development of aptamers offers a new opportunity to overcome the difficulties of traditional methods for detecting toxins and reduce the risk of seafood and water contamination caused by toxins. Numerous aptamers have developed into novel detection methods for toxins^14–16^. However, the interaction mechanism between aptamers and targets still needs further exploration for the therapeutic application of aptamers. In our previous work, we obtained the aptamer(GO18-T-d)^15^ of GTX1/4 and analysed the binding mechanism of GTX1/4 and GO18-T-d by a series of molecular modeling programs^17^. In addition, we also obtained the aptamer P-18S2 that could bind to PTX with a high KD of 0.93 nM^10^ by SELEX and Biolayer interferometry (BLI) which was a real-time optical analytical technique for measuring interaction between biomolecules^18^. In this study, we designed a series of molecular modeling programs in order to explore the further binding mechanism between P-18S2 and PTX and further optimized aptamers for the therapeutic application in the future.

## Results

### Analysis of docking

**Figure 1:**
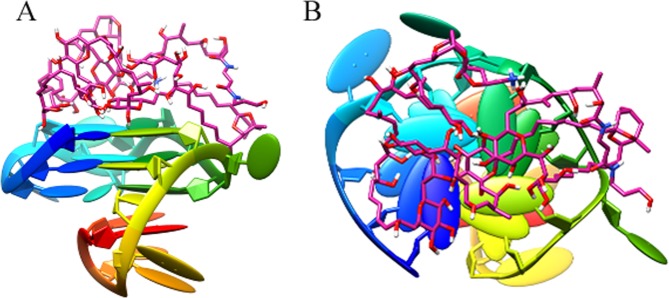
Three dimensional side view (**A**) and top view (**B**) of the top-ranking docked conformations between P-18S2 and PTX.

After modeling and optimization, the 3D structure of DNA P-18S2 with G-quadruplex structure was used as the receptor in the docking. The combination of receptor and ligand was evaluated by the Etotal, Eshape and Eforce in the docking results. The electrostatic energy, Eforce, steric complementarity score and Eshape were combined to give a total energy (Etotal) for the complex (in kJ/mol units)^19^. In our Hex interaction simulation, the lower value of Etotal resulted in more stable combination between DNA and PTX. As was shown in the Table S1, the total calculated interaction energy was listed, which showed the best energy rank results. The best observed interaction energy and steric complementarity score of binding between DNA P-18S2 and PTX were calculated to be −509.9 kcal mol^−1^ and −453.4 kcal mol^−1^ respectively, which indicated that the combination of P-18S2 and PTX was extremely stable, and matched well in the shape (Fig. 1). Furthermore, the electrostatic energy between P-18S2 and PTX was extremely low, which represented a high value of interaction energy and stable combination between P-18S2 and PTX.

**Figure 2:**
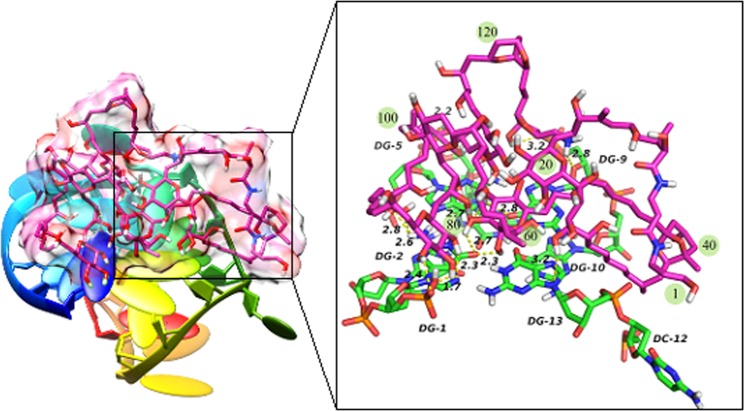
Three dimensional view of the interaction between P-18S2 and PTX.

In order to understand the interaction between DNA and ligands, deep analysis was performed on the top-ranking docked conformation of complex. As was shown in the Fig. 1, P-18S2 and PTX eventually formed stable complex structure during mutual induction, the PTX inserted into the docking sites at the top of P-18S2. Quantitative analysis of H-bond distance was performed, which was shown in Fig. 2. It was noted obviously that there were six H-bonds from bases of G1, G5, G9 and G13 between P-18S2 and PTX, which increased the stability of complex to a great extent (Fig. 2). Probably, due to the strong electrostatic interaction and hydrogen-bonds, P-18S2 showed the obstruction of the binding activity between sodium channel proteins and palytoxin.

### Analysis of molecular dynamics simulation

**Figure 3:**
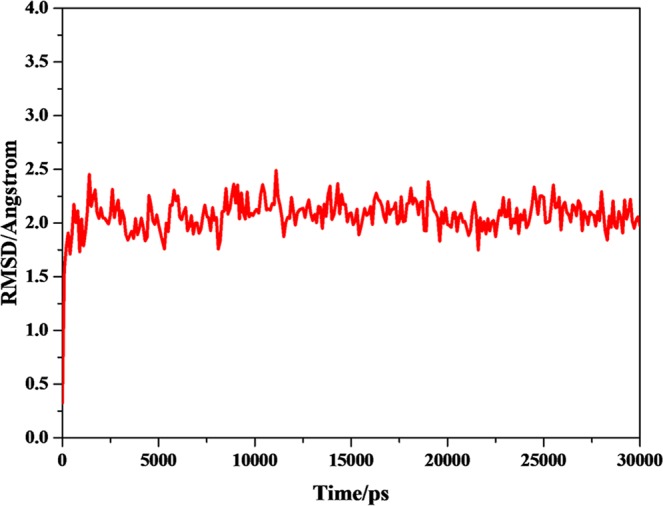
RMSD of CA atoms versus simulation time.

**Figure 4:**
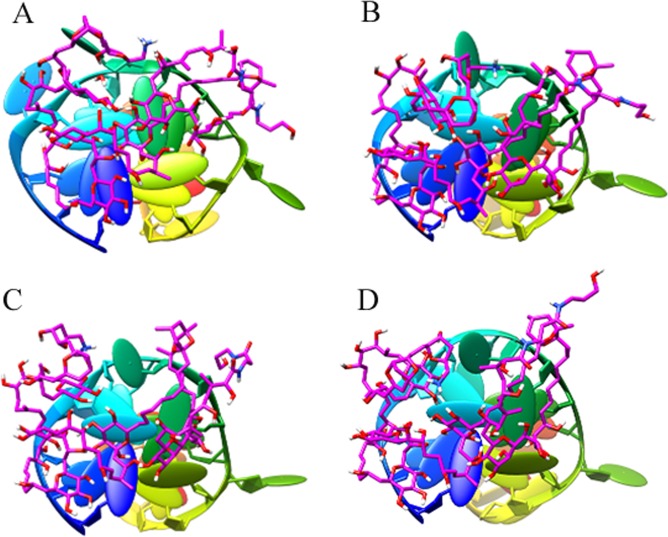
Top-view of the conformations of complex P-18S2-PTX versus MD simulation time. (**A**: 0 ns; **B**: 0 ns; **C**: 20 ns; **D**: 30 ns).

**Figure 5:**
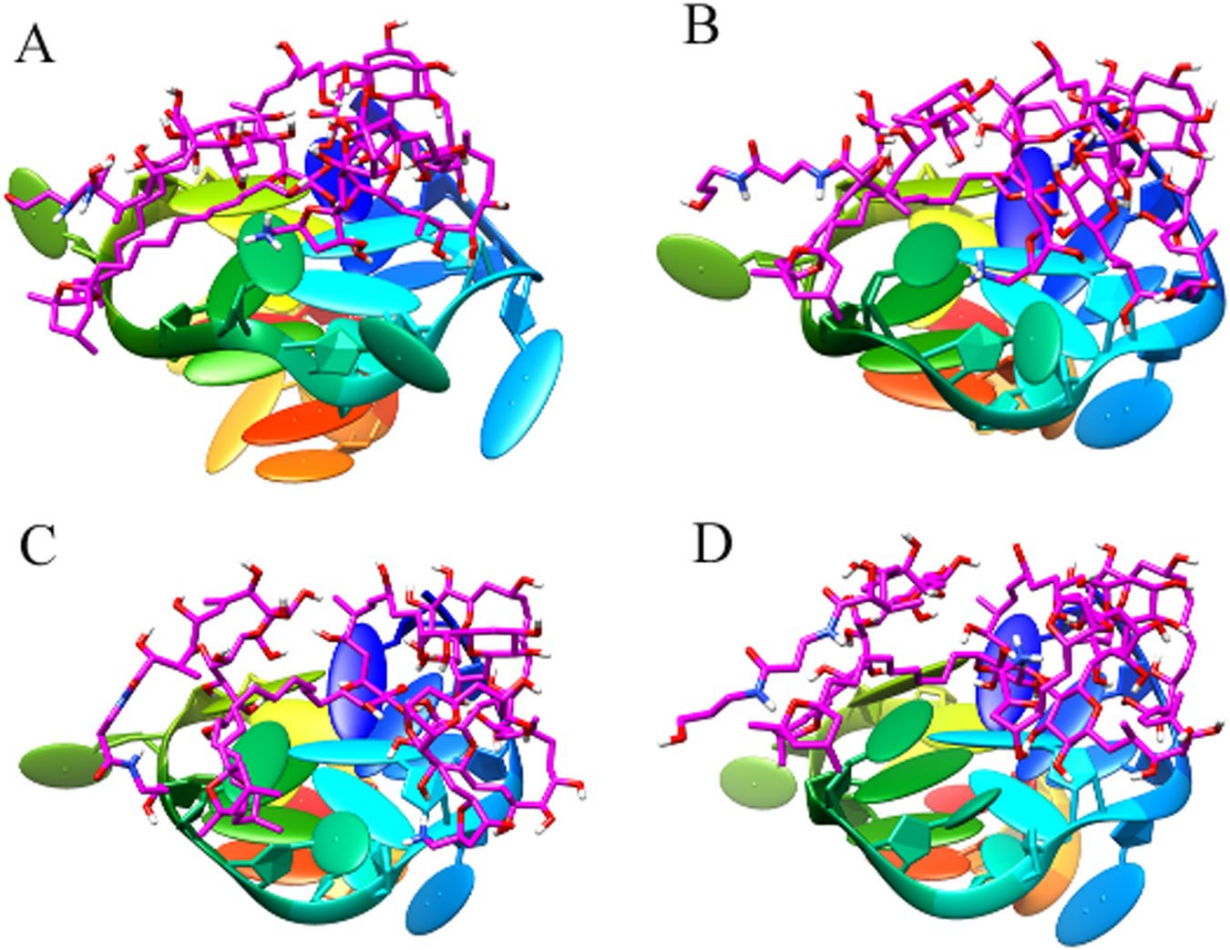
Oblique-view of the conformations of complex P-18S2-PTX versus MD simulation time. (**A**: 0 ns; **B**: 10 ns; **C**: 20 ns; **D**: 30 ns).

**Figure 6:**
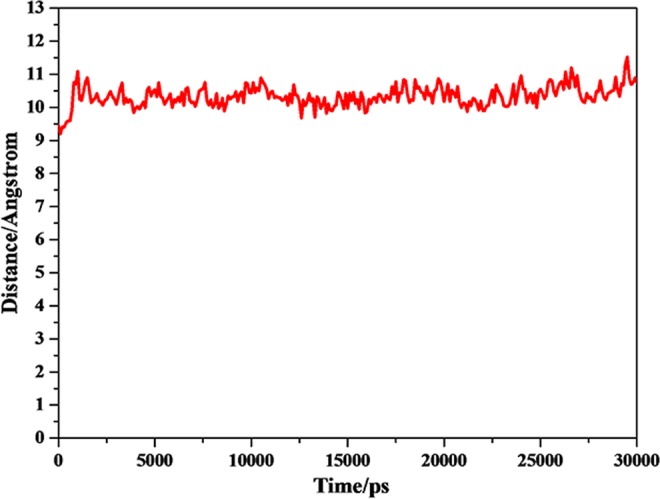
Mass distance between P-18S2 and PTX versus simulation time.

**Figure 7:**
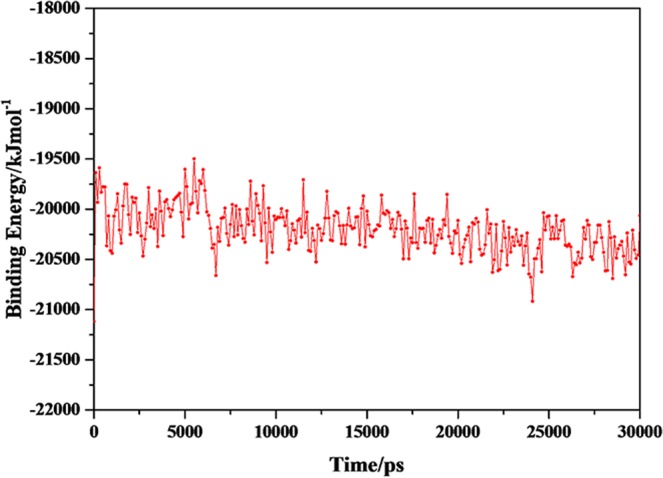
Binding energy of complexes versus simulation time.

A common way to analyze the structural stability of biomacromoleculars in MD simulation is to monitor the root-mean-square deviation (RMSD) from the initial simulation structure. The RMSD of the complexes in this study were shown in Fig. 3. It was clear from plots that the complexes of P-18S2-PTX reached an equilibrium state with fluctuation of 2 Å after 2 ns. Furthermore, in 30 ns of simulation the structures were inconsiderably distinct from the initial structures that were employed as the starting point of the simulations (Figs 4 and 5), indicating that the complexes were stable. Without isolation from each other, the conformation of complexes with different time from 0 ns, 10 ns, 20 ns and 30 ns were similar.The mass distance of G-quadruplex structure and PTX had also demonstrated it (Fig. 6). It was clear that there was no obvious fluctuation in the plots of mass distance between DNA and PTX versus simulation time. In addition, the binding energy (Fig. 7) of complex P-18S2-PTX kept equilibrated, indicating that there was no separation between the DNA and PTX, and they combined steadily. If there was dissociation, the energy would fluctuate severely. The absence of fluctuation showed the strong non-bond interaction between DNA and PTX, which made the complex more stable. Consequently, the high affinity between PTX and DNA P-18S2 blocked the binding between sodium channel protein and PTX.

**Figure 8:**
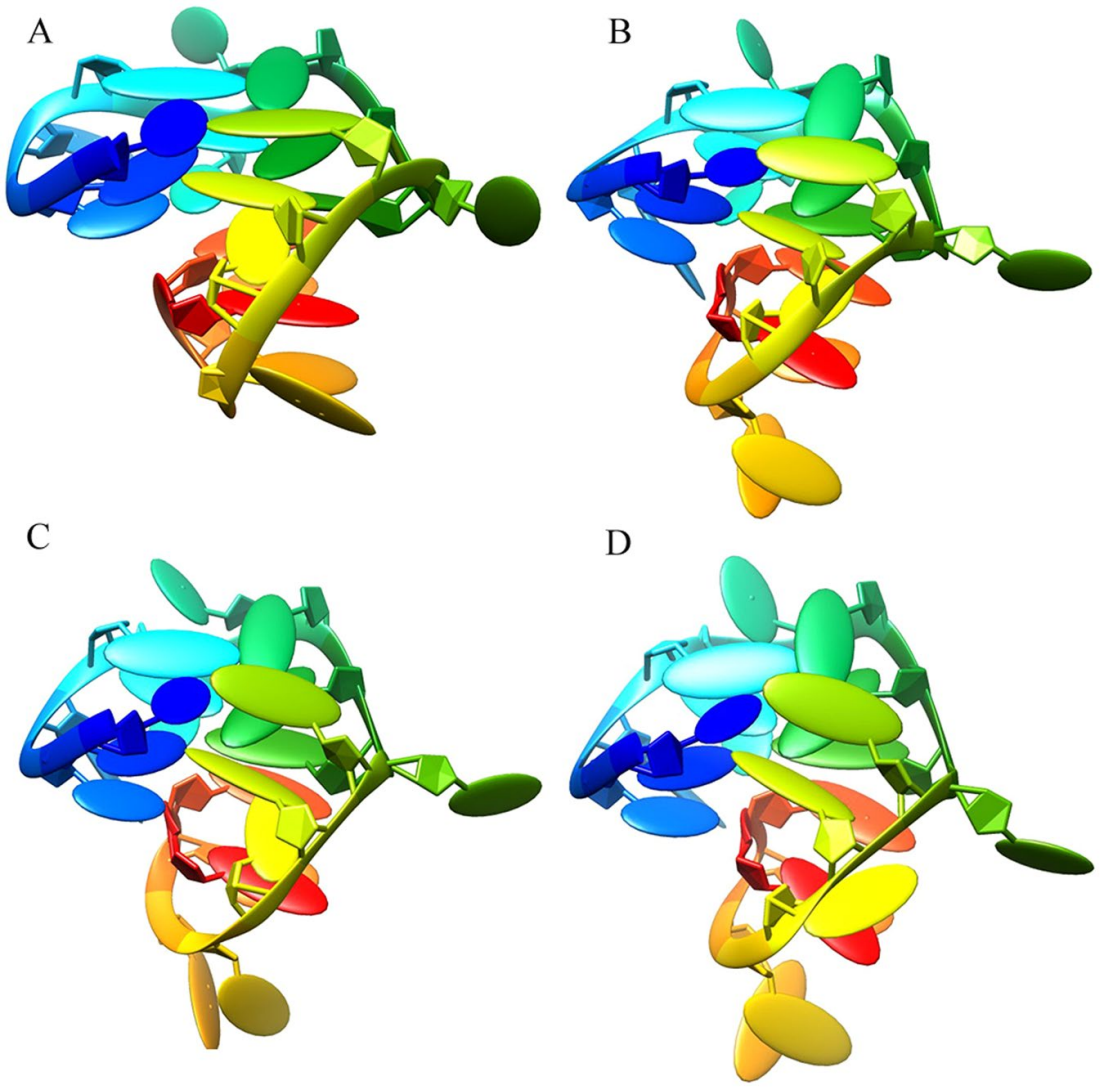
Oblique view of the conformations of G4 structure in P-18S2 during MD simulation (**A**: 0 ns; **B**: 10 ns; **C**: 20 ns; **D** 30 ns).

**Figure 9:**
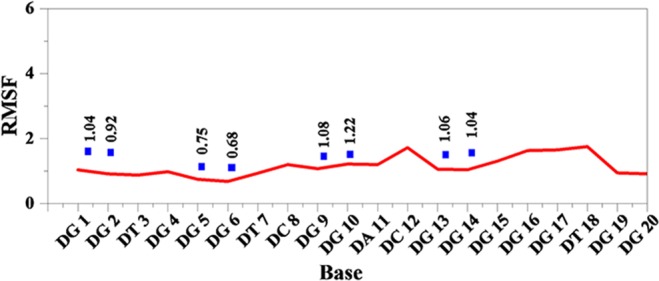
RMSF of residues of complex versus simulation time.

The stability of G-quadruplex structure was analyzed as well. As was shown in Fig. 8, the G-quadruplex structure of DNA P-18S2 exhibited little fluctuation in the simulation, indicating that DNA P-18S2 with G-quadruplex structure had strong intramoleculars interaction, which led to the stability of G-quadruplex structure. As a result, RMSF (Fig. 9) of the key bases of G-quadruplex structure, such as G1, G2, G5, G6, G9, G10, G13 and G14 in P-18S2, showed a small value less than 2 Å. So it could be concluded that DNA with G-quadruplex structure kept steady in the progress of MD simulation, which led to the stable combination between PTX and DNA. After quantitative analysis, it was found that the RMSF of the key bases of G-quadruplex structure (G1, G2, G5, G6, G9, G10, G13 and G14 with a value of 1.03, 0.91, 0.74, 0.68, 1.07, 1.21, 1.05 and 1.04 Å respectively) from P-18S2 was extremly low. That was why P-18S2 showed high activity for blocking the binding between PTX and sodium channel protein.

**Figure 10:**
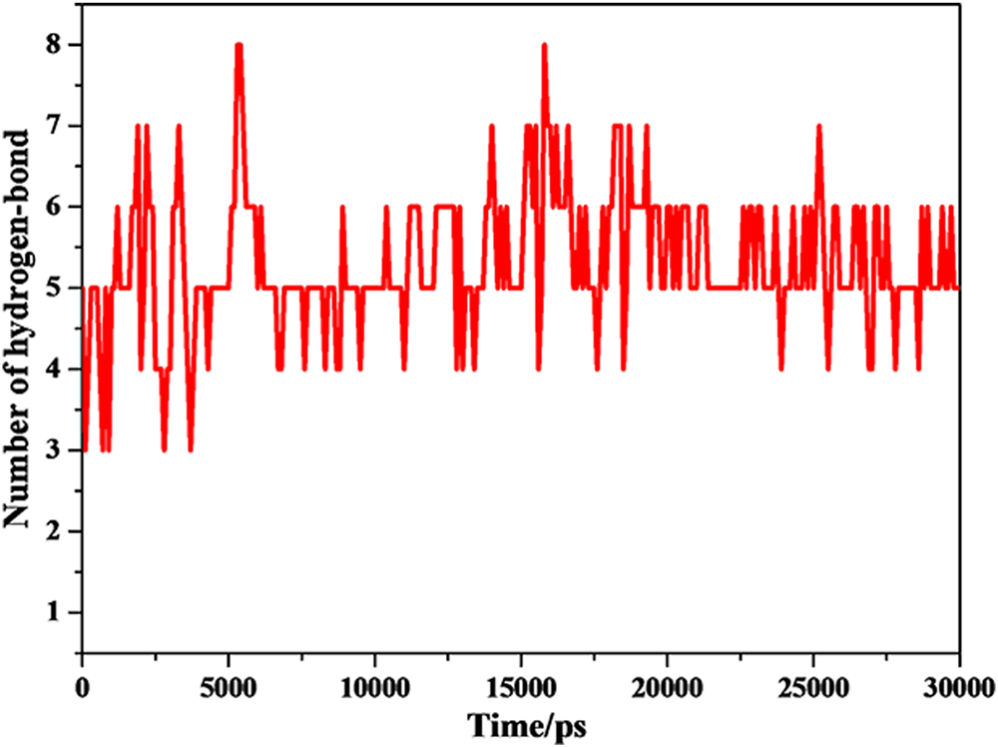
Hydrogen bonds between P-18S2 and PTX versus simulation time.

**Figure 11:**
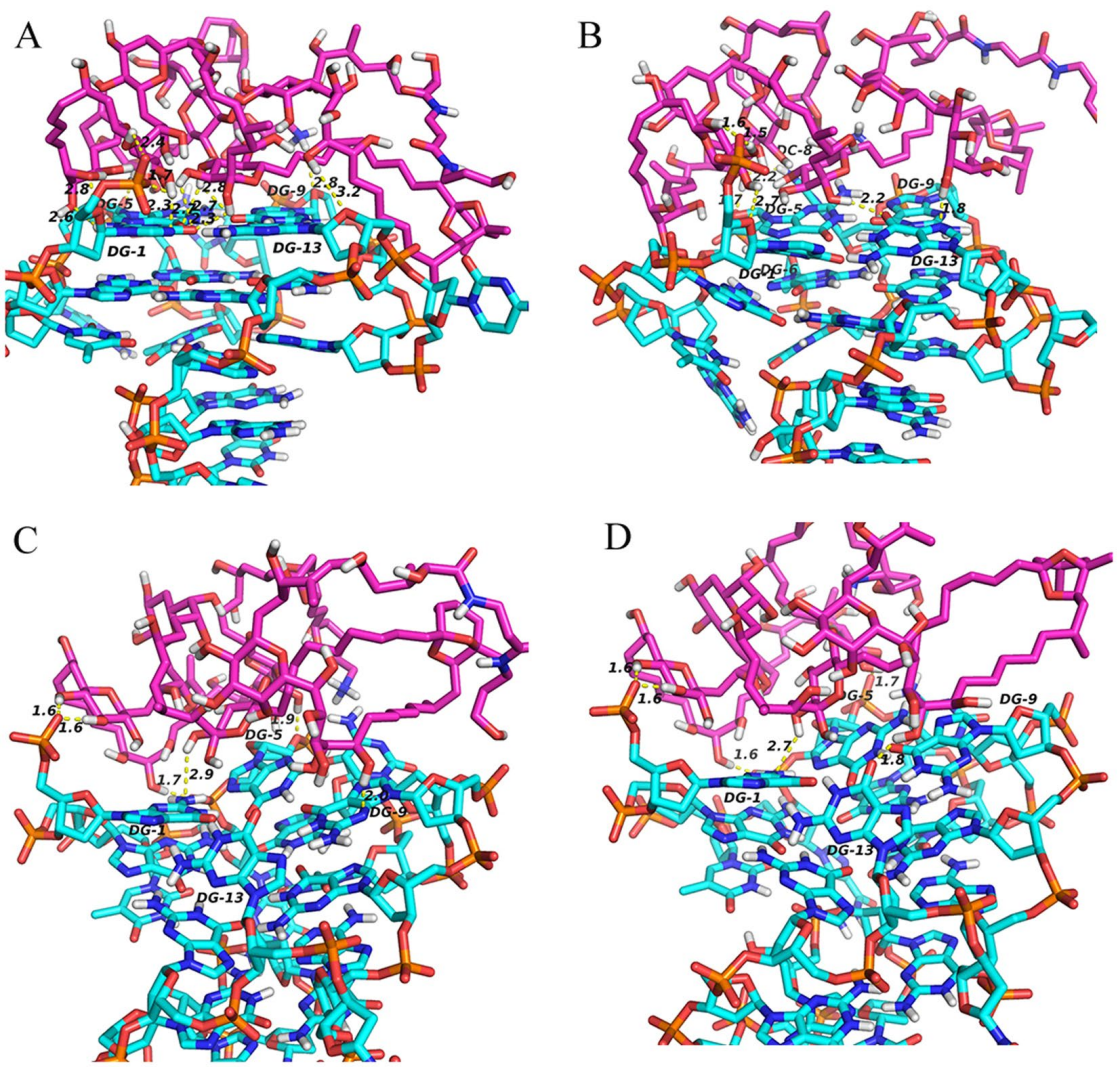
3D-view of hydrogen bonds interaction between P-18S2 and PTX versus simulation time.

In order to give a new insight into the binding of DNA with PTX during the MD simulation, the hydrogen bonds were monitored(Fig. 10). The plots of the hydrogen bonds between DNA and PTX indicated that the DNA combined with PTX stably by the strong hydrogen bond interaction. In the process of dynamics simulation, the hydrogen bonds of the complex always existed. Some hydrogen bonds existed only for a period of time, named dynamic hydrogen bonds, while others, called static hydrogen bond, persisted through the MD simulations. The analysis of the dynamic behaviour of hydrogen bonds was performed to explore the blocking mechanism of DNA for the combination of PTX and sodium channel protein. The 3D structure of the interaction of DNA P-18S2 with PTX system were visualized in Fig. 11. It was found that there were both static hydrogen bonds and dynamic hydrogen bonds between DNA P-18S2 and PTX during the dynamics simulation. As was shown in Fig. 11, there were three static hydrogen bonds and eighteen dynamic hydrogen bonds between P-18S2 and PTX, made the binding of P-18S2 and PTX more stable and more active to block of the binding of PTX to sodium channel protein.

**Figure 12:**
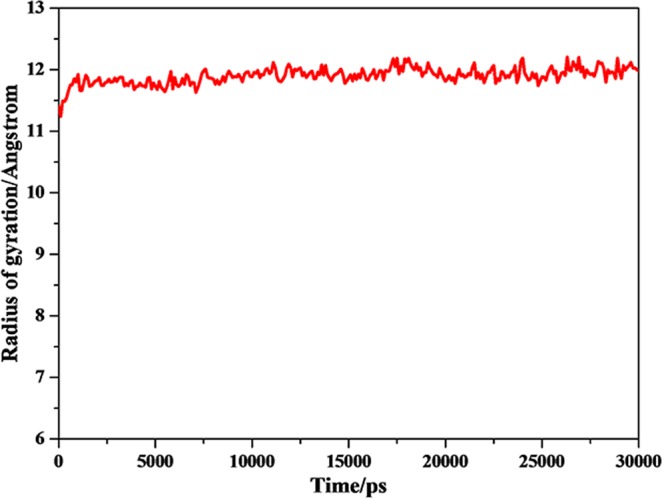
Radius of gyration of complex versus simulation time.

Strong hydrogen bonds interaction promoted the stability of the complexes. The gyration radius of trajectory diagram also proved this point. The gyration radiu of biomacromolecular was a sign of stability in the process of dynamics simulation. As was shown from gyration radius of trajectory diagram from the complex (Fig. 12), gyration radius was stable after 5 ns and the fluctuation value was negligible. Thus, the gyration radius of complex further validated the stable combination between G-quadruplex DNA (P-18S2) and PTX.

### Affinity of truncation of P-18S2 and PTX

The Biolayer interferometry (BLI) results showed that PTX interacted with successively truncated sequence P-18S3,P-18S4, P-18S5, P-18S6, P-18S7, P-18S8 with a *K*_d_ (nM) of 3.14, 2.13, 0.87, 0.81, 2.93, 2.62, respectively, and P-18S2 with a *K*_d_ of 1.09 nM(Table. S2 and Fig. S1). These data were very close to the previous result(0.93 nM)^10^, and such data were not observed in the corresponding blank or negative controls, which demonstrated that there was no significant difference between truncated P-18S2 and primary P-18S2 when they binded PTX. In addition, this result further verified the reliability of computer simulation and defined the most core binding site between P-18S2 and PTX.

## Discussion

Molecular simulation is a standard protocol to study molecular interaction, especially for macromolecules, such as protein, that has been known since the past decades. However, for small molecules such as aptamers, RNA aptamers are a bit easier because structure prediction softwares such as RNA Composer in Poland is free on the web, but DNA is comparatively difficult due to a lack of free high-quality 3D DNA structure prediction software and the flexibility of ssDNA in solution. Therefore, in order to further study the complex structure and interaction between P-18S2(ssDNA) and PTX, a series of molecular modeling programs are designed, including modeling, traditional dynamics simulation and molecular docking. At first, we predicted the stable structure of P-18S2 with high probability through online free structure prediction software QGRS. And then, in the existing structure library, the crystal structure similar to the previous prediction was searched, used as a template and docked with the PTX. Therefore, the simulation deviation caused by the flexibility of the ssDNA could be largely avoided, and the reliability and accuracy of the simulation result could be improved. Certainly, we have decreased the diversity of the DNA aptamer structure when reducing the effect of aptamer flexibility. Therefore, our research also has limitations, and we hope to further perfect the study of DNA aptamer structure through sustained efforts in the future.

Modeling results revealed that the structure of P-18S2 was a DNA G-quadruplex. Meanwhile, the 3D structure of PTX with the lowest total energy after equilibrium was selected for the subsequent simulation. Then, based on the DNA G-quadruplex structure, the combination model of P-18S2 and PTX was predicted, and docking results showed that P-18S2 and PTX eventually formed stable complex structure during mutual induction. PTX could combine steadily at the groove’s top of P-18S2 model by strong hydrogen-bonds and electrostatic interaction. This paper further refine the research method based on our previous study^17^, the flexibility of the target structure is evaluated and the biological experiments are used to confirm the veracity of the simulation results. When binding to PTX, the affinity of 6 optimized aptamers by simulation was respectively compared with the primary P-18S2, and the BLI results showed no significant difference. Thus, integrating experimental results and simulation results, reasonable interpretation is provided for the high affinity and specific binding between P-18S2 and PTX, and the simulated binding sites are firmly proved as the core sequence of P-18S2 by the detection of binding affinity. Finally, the P-18S2 was further optimized for subsequent bioactivity studies *in vivo*. In summary, this study established a feasible model for aptamer (ssDNA) and its target in molecular structure analysis and special induced fit mechanism, and laid the foundation for the study of aptamer in molecular diagnostics and therapeutic applications.

## Methods

### G-quadruplex model generation

The structure prediction of P-18S2 was performed with QGRS(*quadruplex forming G-rich sequences*, http://bioinformatics.ramapo.edu/QGRS/index.php). Minimum G-Group size was settled as 2. There were 56 possible structures for P-18S2 in forming G-quadruplex structure, the top 10 results of QGRS prediction were showed in Table S3, with a maximum G-Score of 21. Thus, the possibility was high that P-18S2 formed G-quadruplex structure.

**Figure 13:**
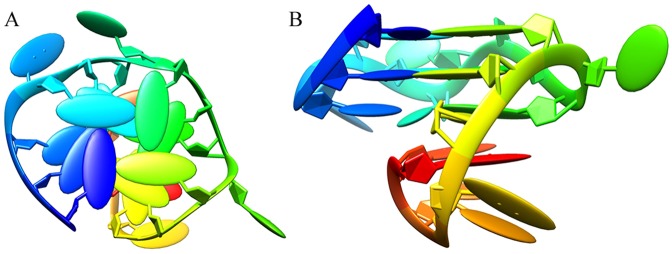
The 3D model of P-18S2 with G-quadruplex structure, top view at left and side view at right.

Then, 193 entries of quadruplex DNA structures were searched in the Nucleic Acid Database (NDB)^20^. Unfortunately, this list did not include all G-quadruplex structures. The Protein Data Bank (PDB)^21^ was searched as well for entries containing the words “quadruplex” and the list was filtered by visual inspection. The sequences extracted form each chain in the corresponding PDB files were aligned to P-18S2. The 2LXQ^22^ was found to exhibit corresponding intrastrand G-quadruplexes and similar size with P-18S2. In addition, the arrangement of guanines in 2LXQ were similar with P-18S2 to a certain extent. Based on the atom model of 2LXQ, the 3D model of P-18S2 with G-quadruplex structure was generated by Discovery Studio 2.5 Client (http://accelrys.com/products/discovery-studio) with the nucleic acids substitution, insertion and deletion. Considering the optimal coordination geometry, conformation of a few nucleic acids were adjusted manually within the allowed range. Then, the optimization of model was performed at the high performance computing facility with the YASARA package^23,24^, using the Amber 14 force field^25^, and the water model was TIP3P. The temperature coupling of the model system was ascertained by Berendsen thermostat method, while the manometer method was used for pressure coupling^26^. Besides, the initial structure was placed in a rectangular analog box with periodic boundaries, and the solvent was water. For the operation of optimization in the simulated water condition, the backbone was firstly fixed and the side chain was optimized 5,000 steps, and then, the whole structure was majorized 5,000 steps. Finally, the optimized 3D-struture of P-18S2 with G-quadruplex structure was generated (Fig. 13). After optimization, quantitative analysis was performed to the G-quadruplex structure of P-18S2. The results showed that P-18S2 formed stable G-quadruplex structure due to strong hydrogen-bonds interaction among G1, G5, G9, G13 and G2, G6, G10, G14 in P-18S2 (Fig. S2).

### Docking

**Figure 14:**
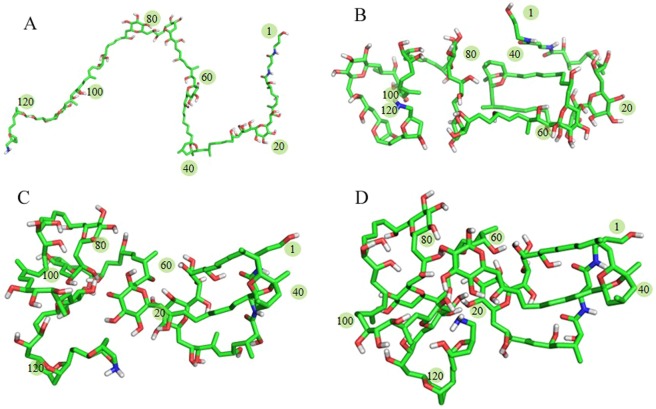
Conformation change of the PTX in the process of dynamic simulation. (**A**: 0 ns; **B**: 10 ns; **C**: 20 ns; **D**: 30 ns).

Firstly, PTX was obtained from PubChem Compound database with accession no. 45027797 (Fig. S3). The PTX molecule was optimized using MM2 method. Due to large size and high molecular weight, PTX showed high flexibility and lots of possible conformations. Thus, it was unreasonable to perform the docking using PTX from the database directly. The solution to these problems was to first obtain the equilibrium structure of PTX in the solvent and use it for molecular docking. Only in this way could we scientifically investigate the interaction between PTX and DNA P-18S2. In this work, PTX in KCl aqueous solution was performed in 30 ns dynamics simulation using GAFF force field^27^, then repeated three times. It was found that in the process of dynamics simulation, the PTX tended to constrict in aqueous solution and reached an equilibrium state (Fig. 14). The 3D structure of PTX after equilibrium was output and optimized. Subsequently, the total energy was calculated, and the structure with lowest energy was selected for the subsequent simulation. Then, the number of rotatable bonds of ligands was set up in AutodockTools^28^. Finally, PTX was output and saved as the ligand.

For DNA-ligand docking, 3D structure of P-18S2 was obtained from the modeling and initialized as receptor molecule with AutodockTools. Subsequently, the receptor was endowed with AD atomic type, and hydrogen and charge were added, followed by the mergence of nonpolar hydrogen. The binding sites of P-18S2 recognizing ligand were obtained based on crystal structure of DNA-ligand complex with G-quadruplex structure^29–33^, which proved that the binding site of DNA with G-quadruplex structure was at the groove’s top. In addition, the experiment suggested that the ligand could induce the formation of G-quadruplex structure. Therefore, as demonstrated in our previous work^17^, the binding site of P-18S2 with G-quadruplex structure was at the groove’s top (Fig. S4).

Finally, the molecular docking analysis was carried out using Hex 8.0^34^. Without the specified binding group, rotation of receptor and ligand was allowed in 180 degree. The shape and electrostatic interaction were also considered. The maximum number of docking was revised as 200 and cluster analysis was made, while the default parameter settings were retained for others. For each of the docking cases, the lowest energy conformation, according to the Hex scoring function, was selected as the binding mode. The output from Hex was rendered with Chimera^35^ and PyMol program^36^.

### Molecular dynamics simulation

The Molecular dynamics (MD) simulation was carried out based on the complex which obtained from docking in Hex program. All simulations were performed using the molecular dynamics program YASARA and the amber 14 force field^37^. The complex using in the simulation came from molecular docking with hydrogen generated by the YASARA program. All simulations were carried out with an integration step of 1 fs and coordinates of the simulation model were recorded per 1 ps. The starting structures were immersed in a periodic rectangular simulation cubic cell of KCl aqueous solution. The box dimensions were chosen to provide at least 10 Å buffers of solvent molecules around the solute.

The fully soluble system was then subjected to 5000 steps steepest descent, minimization runs to remove clashes between atoms. An 80 ps position restrained MD simulation was performed for each system at constant pressure (1 atm) and temperature (300 K). The temperature and pressure were kept constant during the simulation. Temperature coupling was done using the Berendsen thermostat with a temperature coupling constant of 0.1 ps, while the manometer method was used for pressure coupling, with a reference pressure of 1 atm. A particle mesh Ewald scheme^38,39^ was used to calculate the long range electrostatic interactions with a 10 Å cutoff for the real space. A cutoff of 14 Å was used for the van der Waals interactions (Lennard-Jones terms). Translation and rotation corrections were enabled during MD simulations to ensure that structures in trajectory were well superimposed, which was convenient for the structure analysis. The chemical bond lengths involving hydrogen atoms were fixed with SHAKE algorithm^40^.

By the time of 1 ns, the simulation system reached an equilibrium state; thus, the system was subjected to conventional MD (CMD) simulation for 30 ns. All calculations were performed on the MolDesigner molecular simulation platform.

### Determination of affinity by BLI

We were surprised to find that P-18S2 could be further truncated and optimized by simulating and docking. Therefore, the affinity between the successively truncated sequence (P-18S3~P-18S8) and P-18S2 and PTX were determined respectively. The super streptavidin-coated (SSA) biosensor was used for the immobilization of 7 sequences onto the Biolayer interferometry (BLI) aptasensor. Those aptamers were analyzed for association over 2 min with PTX (5 uM) and dissociation over 2 min, along with a blank sample containing only binding buffer for reference and a random sequence was used as a negative control.

## Supplementary information

supplementary material

The binding dynamic image of Aptamer(P-18S2) and toxin(PTX)
