# Supplementary material for: Study of the binding mechanism of aptamer to palytoxin by docking and molecular simulation
Source: Sci Rep. 2019 Oct 29;9:15494. doi: 10.1038/s41598-019-52066-z (PMC6820544; doi:10.1038/s41598-019-52066-z)
Supplement: Supplementary file 1 — supplementary material [file 41598_2019_52066_MOESM1_ESM.pdf]

Study of the binding mechanisms of aptamer to palytoxin by docking and molecular simulation

Bo Hu[1,2+], Rong Zhou[1,2+], Zhengang Li[1,2+], Shengqun Ouyang[1], Zhen Li[1], Wei Hu[3], Lianghua Wang[1]*, Binghua Jiao[1,2]*

[1]*Department of Biochemistry and Molecular Biology, College of Basic Medical, Second Military Medical University, Shanghai 200433, China.*

[2]*Marine Biological Institute, College of Marine Military Medicine, Second Military Medical University, Shanghai 200433, China.*

[3]Chengdu FenDi Technology Co., Ltd, Chengdu 610041, China.

* Corresponding author

E-mail addresses: bhjiao@smmu.edu.cn (Binghua Jiao),

lhwang@smmu.edu.cn (Lianghua Wang).

+these authors contributed equally to this work

Table S1 Docking results between DNA with G-quadruplex structure and palytoxin

Cluster	Etotal[a]/kcal▪mol^{-1}	Eshape[b]/ kcal▪mol^{-1}	Eforce[c]/ kcal▪mol^{-1}	RMS[d]
1	-509.9	-453.4	-56.5	-1.00
2	-376.7	-423.5	46.8	-1.00
3	-351.8	-412.3	60.5	-1.00
4	-335.3	-330.1	-5.1	-1.00
5	-318.7	-364.7	46.0	-1.00
6	-302.7	-336.5	33.8	-1.00
7	-286.2	-361.0	74.9	-1.00
8	-266.2	-341.0	74.8	-1.00
9	-240.0	-330.4	90.4	-1.00
10	-194.8	-335.1	140.4	-1.00

[a] The total calculated interaction energy; [b] The original Hex steric complementarity score; [c] The electrostatic energy;[d] Root-mean-square deviation of the top-scoring docking orientation

Table S2 The K_d values of aptamers combined with PTX

ID	sequence	K_d
P-18S2	GGTGGGTCGGACGGGGGTGG	1.09nM
P-18S3	GGTGGGTCGGACGGGGGTG	3.14nM
P-18S4	GGTGGGTCGGACGGGGGT	2.13nM
P-18S5	GGTGGGTCGGACGGGGG	0.87nM
P-18S6	GGTGGGTCGGACGGGG	0.81nM
P-18S7	GGTGGGTCGGACGGG	2.93nM
P-18S8	GGTGGGTCGGACGG	2.62nM

Table S3 The results of P-18S2 from QGRS prediction

Position	Length	QGRS	G-Score
1	14	GGTGGGTCGGACGG	19
1	14	GGTGGGTCGGACGG	21
1	15	GGTGGGTCGGACGGG	19
1	15	GGTGGGTCGGACGGG	20
1	16	GGTGGGTCGGACGGGG	18
1	16	GGTGGGTCGGACGGGG	14
1	16	GGTGGGTCGGACGGGG	19
1	16	GGTGGGTCGGACGGGG	15
1	16	GGTGGGTCGGACGGGG	15
1	17	GGTGGGTCGGACGGGGG	17

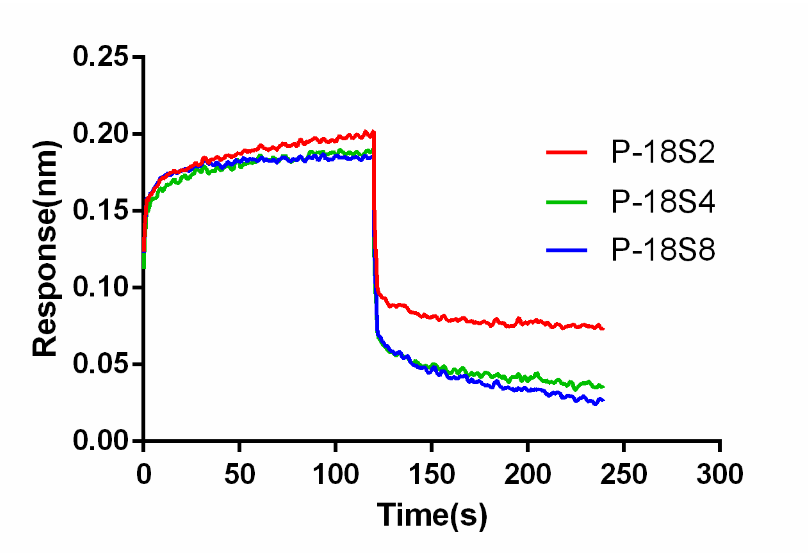

Fig S1 The red, green and blue lines respectively represent the interaction curve of aptamer P-18S2, P-18S4, P-18S8 with PTX (5 uM).

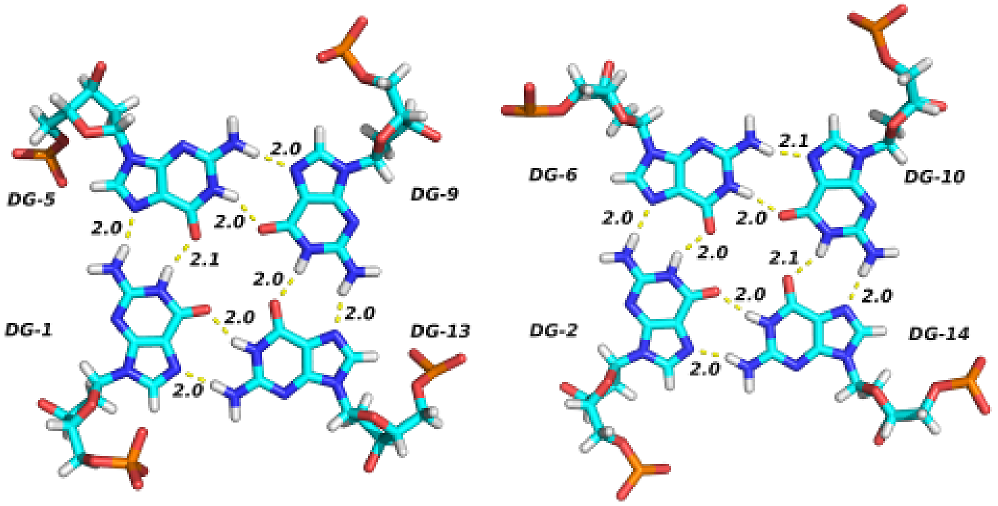

Fig S2 The G-quadruplex structure and intramolecular hydrogen bonds of P-18S2

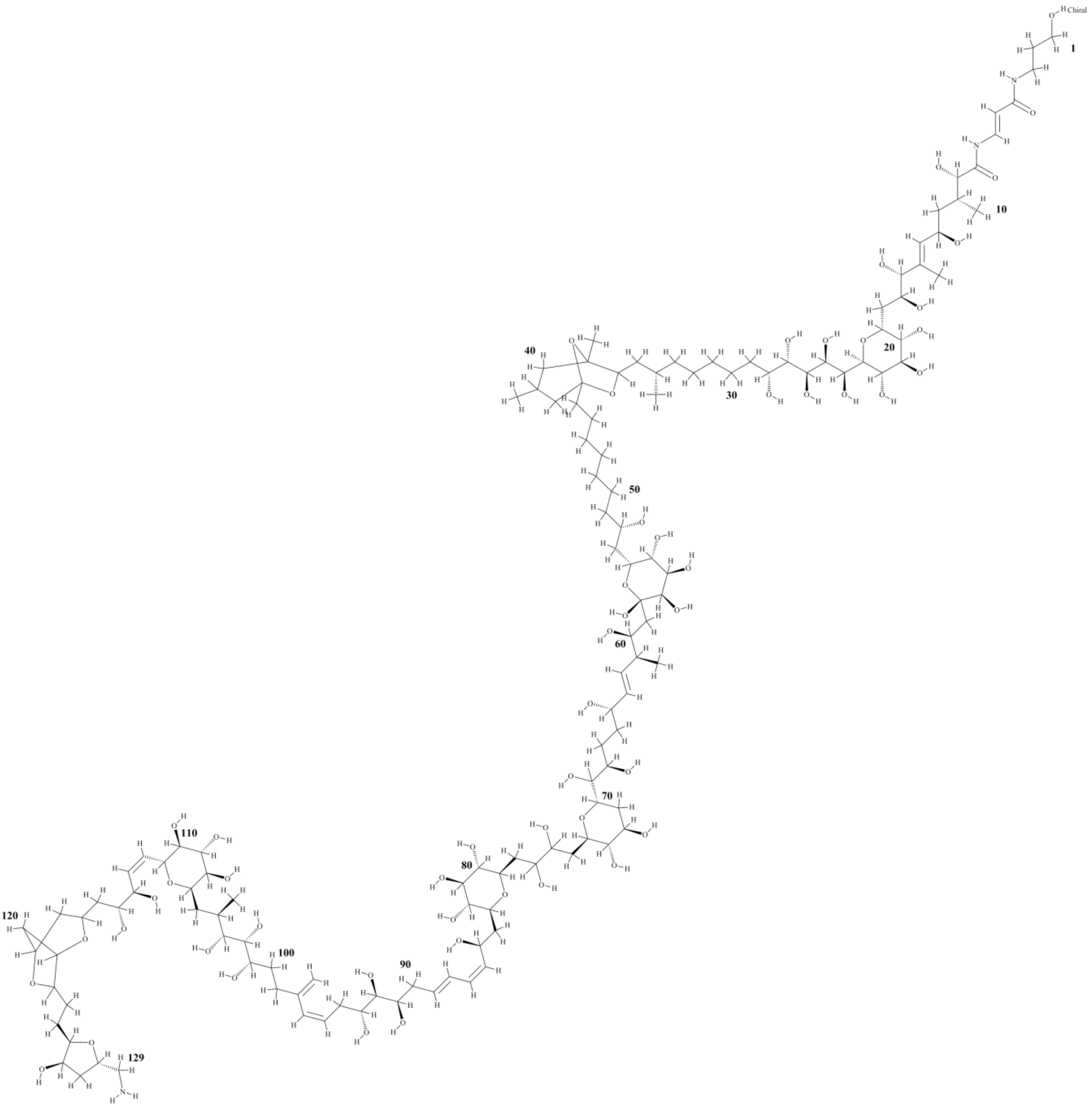

Fig S3 Schematic representation of palytoxin

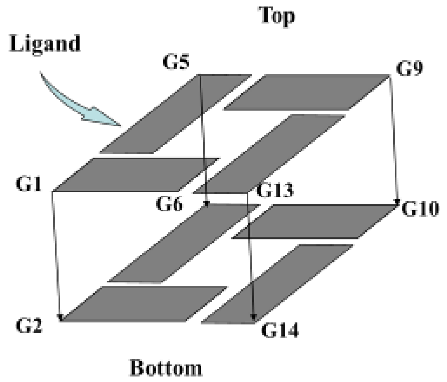

Fig S4 The binding site of P-18S2 with G-quadruplex structure
